# An Observatory for the *MET* Oncogene: A Guide for Targeted Therapies

**DOI:** 10.3390/cancers15184672

**Published:** 2023-09-21

**Authors:** Dogus M. Altintas, Paolo M. Comoglio

**Affiliations:** IFOM ETS—The AIRC Institute of Molecular Oncology, 20139 Milano, Italy; dogus.altintas@ifom.eu

**Keywords:** *MET* alterations, cancer progression, invasive growth, therapeutic targeting

## Abstract

**Simple Summary:**

The *MET* gene encodes a receptor critical for cell growth and repair. It plays diverse roles in processes like organ development, wound healing, and blood vessel formation. Genetic alterations in *MET* contribute to cancer progression, enabling tumor spread and resistance to treatment. Scientists are studying how to target MET to treat cancer. Despite progress, the complexity of MET’s functions in cancer challenges our understanding. This review explores recent discoveries about MET in cancer, its effects, potential therapies, and future directions.

**Abstract:**

The *MET* proto-oncogene encodes a pivotal tyrosine kinase receptor, binding the hepatocyte growth factor (HGF, also known as scatter factor, SF) and governing essential biological processes such as organogenesis, tissue repair, and angiogenesis. The pleiotropic physiological functions of MET explain its diverse role in cancer progression in a broad range of tumors; genetic/epigenetic alterations of *MET* drive tumor cell dissemination, metastasis, and acquired resistance to conventional and targeted therapies. Therefore, targeting MET emerged as a promising strategy, and many efforts were devoted to identifying the optimal way of hampering MET signaling. Despite encouraging results, however, the complexity of MET’s functions in oncogenesis yields intriguing observations, fostering a humbler stance on our comprehension. This review explores recent discoveries concerning *MET* alterations in cancer, elucidating their biological repercussions, discussing therapeutic avenues, and outlining future directions. By contextualizing the research question and articulating the study’s purpose, this work navigates MET biology’s intricacies in cancer, offering a comprehensive perspective.

## 1. Introduction

The human *MET* protooncogene encodes the tyrosine kinase receptor for the hepatocyte growth factor/scatter factor (HGF/SF) [1,2,3]. It is expressed in a broad range of epithelial cells, and the resulting receptor is a 170 kDa transmembrane protein organized in two disulfide bond-linked *α* and *β* subunits of, respectively, 50 kDa and 145 kDa [4,5]. The ligand, SF, is produced and secreted in physiologic conditions by mesenchymal cells close to *MET*-expressing epithelial cells [6,7]. This paracrine effect leads to MET activation by autophosphorylation of the cytoplasmic catalytic domain and recruitment of adaptor proteins, promoting signal transduction. The mitogen-activated protein kinase (MAPK) pathway, Phosphoinositide 3-kinase/Protein kinase B (PI3K/AKT) signaling, and Signal Transducer and Activator of Transcription 3 (STAT3) constitute the primary signal transducers [8,9,10]. MET plays an essential role during embryogenesis (e.g., epithelial to mesenchymal transition, organ development) and the postnatal period (e.g., angiogenesis, organ regeneration, and wound healing, as reviewed in [11]).

The broad range of critical biological responses induced by MET awards this oncogene as a crucial oncogene in tumor progression, enabling cancer cells to survive and escape the hostile primary tumor microenvironment and form distal metastases [12]. *MET* is altered in multiple cancer types and behaves as a pivotal regulator of invasive growth, a complex and intertwined sequence of events including epithelial-to-mesenchymal transition (EMT), scattering, migration, and growth [3,13,14,15,16,17]. Tumor cells harboring such alterations become ‘addicted’ to MET (described in [3]), therefore instituting a tumor cell-specific vulnerability point and justifying targeted therapies. Accordingly, MET inhibition reduces tumor size and impedes metastases in rodent models [12,18,19]. Recently, MET has been suggested as one of the top five proteins to focus on in targeted cancer therapies [20].

However, it should be noted that the path to efficient MET-targeted therapy for patients is long and covered with complications. Despite the presumed ‘addiction’ to MET, many patients do not respond to therapy [12,21,22,23]. Moreover, acquired resistance to MET inhibitors might arise [24,25,26,27].

This review reminds the overall structure of MET and summarizes the observed *MET* alterations in cancer, their impact on invasive growth, and their therapeutic potential. The lessons from the disappointing results of targeted therapies will be investigated to propose more accurate strategies to extend disease-free survival time.

## 2. Structure and Function of the MET Kinase

**Figure 1:**
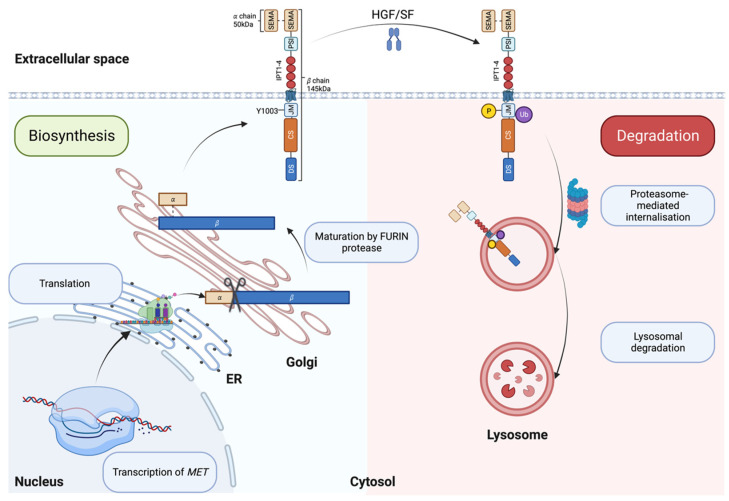
Biosynthesis (**left**, blue box) and degradation (**right**, pink box) of MET. ER: Endoplasmic reticulum; SEMA: semaphorin domain; PSI: plexin–semaphoring–integrin homology domain; IPT: immunoglobulin-like domain; JM: juxtamembrane domain; CS: catalytic site; DS: docking site. Created with BioRender.com (accessed on 5 August 2023).

MET is the receptor tyrosine kinase (RTK) for SF. The translated precursor protein of 175kDa matures through proteolytic cleavage by FURIN protease in the Golgi apparatus (Figure 1, left panel and [28]). The mature protein forms a heterodimer of an extracellular *α* subunit (50 kDa) linked via disulfide bonds to the 145 kDa transmembrane *β* subunit. The extracellular portion of the *β* chain comprises a semaphorin (SEMA) domain, a Plexin–Semaphorin–Integrin (PSI) homology domain, and four immunoglobulin-like IPT domains [29,30]. SEMA and IPT domains are crucial for ligand binding and receptor dimerization, while the PSI domain is essential for the proper maturation of the receptor through its recently described disulfide isomerase activity [31]. The intracellular part of MET is composed of a short juxtamembrane domain (JM), followed by the catalytic site and the docking site for signal transducers. 

**Figure 2:**
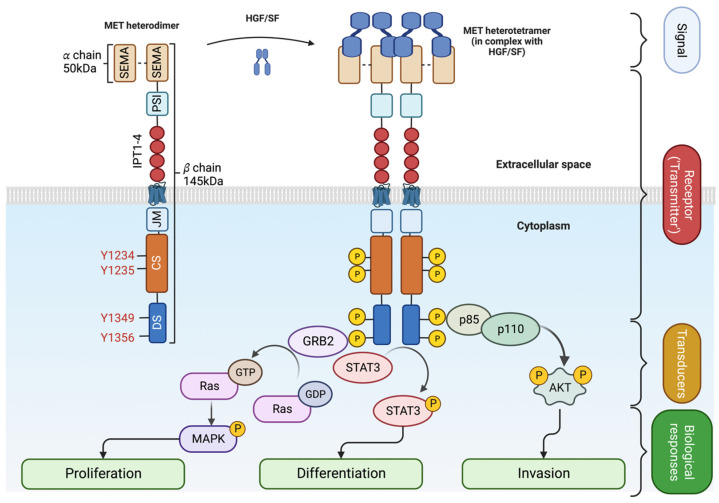
SF-dependent MET activation. Upon ligand binding (HGF/SF), MET heterodimer forms a tetramer [5]. The extracellular signal is transmitted into the cytoplasm, leading to the recruitment of adaptor proteins (GRB2 for MAPK pathway and p85/p110 for AKT) and signal transduction. Created with BioRender.com (accessed on 5 August 2023).

Upon SF binding, the catalytic domain of MET becomes auto-phosphorylated on Tyrosine 1234 and Tyrosine 1235 [32]. The activation of the catalytic site triggers the phosphorylation of two tyrosine on the docking site (Y1349 and Y1356), priming the interaction with Src homology 2 (SH2) domain-containing proteins [10,33,34]. The MET docking site is multifunctional; Y1349 phosphorylation leads to the activation of the PI3K/AKT pathway (migration/survival) while Y1356 phosphorylation activates the RAS/MAPK pathway (proliferation/cell cycle progression) [8,10]. The STAT3 transcription factor is also activated by MET [9]. Like other RTKs, MET transmits the information from the extracellular space to the cytoplasm, generating a multilayered network that activates various biological processes, including migration, growth, and differentiation/stemness (Figure 2 and [35]).

SF/MET interaction induces the phosphorylation of Y1003 within the juxtamembrane domain, allowing the recruitment of the E3 ubiquitin ligase casitas B-lineage lymphoma (CBL), promoting MET monoubiquitylation [36], receptor internalization and lysosomal degradation (Figure 1, right panel and [37]). Accordingly, experimental evidence has shown that Y1003F mutation stabilizes the receptor [38]. The juxtamembrane domain is therefore accepted as a negative regulator of the MET/SF axis [36,39,40,41,42]. 

Proteomics-based studies have been invaluable in understanding conventional RTKs like EGFR, where signaling typically shows rapid activation and deactivation in response to ligand binding [43]. In contrast, MET diverges from this pattern, making it an ‘unconventional RTK’. Unlike conventional RTKs, which generally experience transient phosphorylation followed by rapid deactivation, MET activation remains persistent after ligand binding [44]. In the broader context of RTK signaling, conventional RTKs often follow a swift sequence of activation events involving various cellular components such as MAPK, adaptor proteins, and guanine nucleotide exchange factors [45]. This unique activation profile of MET opens new avenues for future research, particularly to explore the implications of its long-lasting response in both physiology and tumor biology [17].

## 3. *MET* Alterations in Tumors and Their Biological Significance

Recent advances in next-generation sequencing technologies allowed a significant reduction in their cost, ultimately leading to an inflation of publicly available ‘OMICS’ data. Additionally, improvements in the standardization of data curation, analyses, and presentation offered researchers an unprecedented quantity of comprehensive information regarding genetic alterations during cancer onset and progression. In line with this, we developed an auto-updatable ‘MET observatory’ to catalogue genetic alterations of the *MET* gene in cancer. Here, we present some of the features of this observatory; notably, the catalog of alterations results from data collection from The Cancer Genome Atlas (TCGA), Catalogue of Somatic Mutations in Cancer (COSMIC), and ClinVar datasets.

## 4. *MET* Amplification

**Figure 3:**
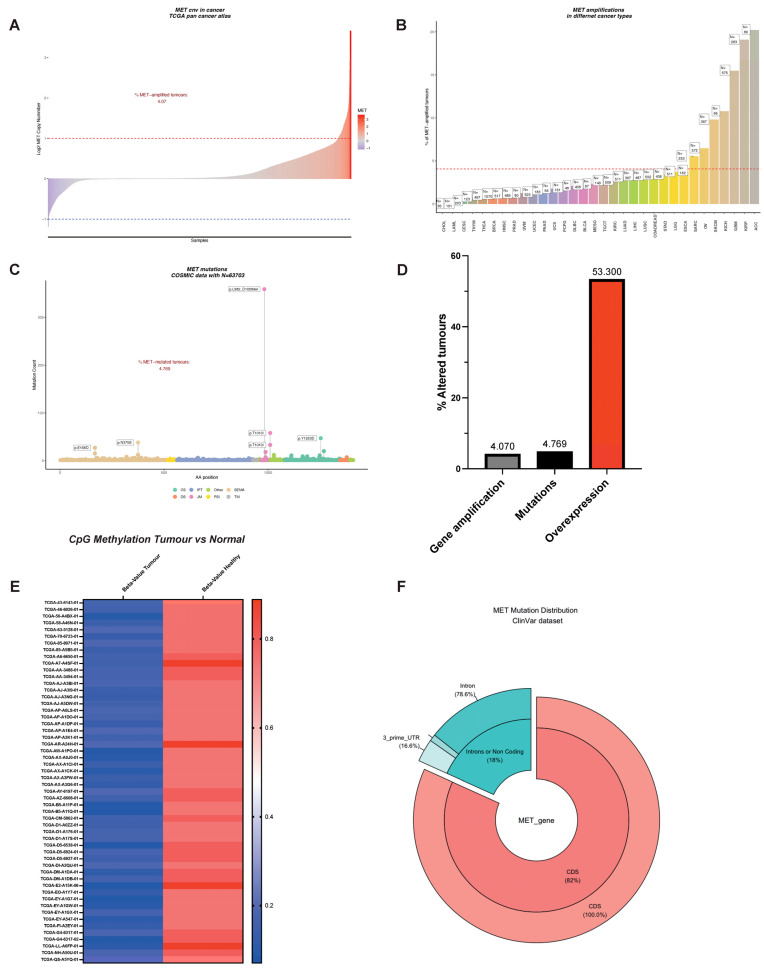
The MET Observatory. (**A**) Copy number variation (CNV) in human tumors (TCGA pan cancer atlas). Dashed lines correspond to a copy lumber variation equal to 2 in absolute values. (**B**) *MET* CNV across tumor types. The dashed line corresponds to the average percentage of CNV in all types of cancer using TCGA abbreviations: ACC (Adrenocortical carcinoma), BLCA (Bladder Urothelial Carcinoma), BRCA (Breast invasive carcinoma), CESC (Cervical squamous cell carcinoma and endocervical adenocarcinoma), CHOL (Cholangiocarcinoma), COAD (Colon adenocarcinoma), DLBC (Lymphoid Neoplasm Diffuse Large B-cell Lymphoma), ESCA (Esophageal carcinoma), GBM (Glioblastoma multiforme), HNSC (Head and Neck squamous cell carcinoma), KICH (Kidney Chromophobe), KIRC (Kidney renal clear cell carcinoma), KIRP (Kidney renal papillary cell carcinoma), LAML (Acute Myeloid Leukemia), LGG (Brain Lower Grade Glioma), LIHC (Liver hepatocellular carcinoma), LUAD (Lung adenocarcinoma), LUSC (Lung squamous cell carcinoma), MESO (Mesothelioma), OV (Ovarian serous cystadenocarcinoma), PAAD (Pancreatic adenocarcinoma), PCPG (Pheochromocytoma and Paraganglioma), PRAD (Prostate adenocarcinoma), READ (Rectum adenocarcinoma), SARC (Sarcoma), SKCM (Skin Cutaneous Melanoma), STAD (Stomach adenocarcinoma), TGCT (Testicular Germ Cell Tumors), THCA (Thyroid carcinoma), THYM (Thymoma), UCEC (Uterine Corpus Endometrial Carcinoma), UCS (Uterine Carcinosarcoma), UVM (Uveal Melanoma). (**C**) *MET* mutations in the protein-coding region and distribution over the MET protein domains. (**D**) Comparison between the incidence of *MET* amplification, mutations, and overexpression. (**E**) B-value of CpG methylation of the *MET* promoter using the *cg22116492* probe in cancer and in non-tumoral tissues. (**F**) Percentage of mutations affecting protein-coding and regulatory regions of *MET* mRNA (ClinVar).

An oncogene can be defined as an entity that can transform cells by conferring some attributes of a cancer cell. *MET* is an excellent example of an oncogene because of its critical involvement in cell migration, metastasis, and cell survival. Unsurprisingly, *MET* is altered in many cancers, including but not limited to non-small cell lung cancer (NSCLC), lung squamous carcinoma, gastric cancer, colorectal adenocarcinoma, melanoma, gliomas, and renal cancer [46,47,48,49,50,51]. One of the most frequently observed molecular aberrations involving *MET* is gene amplification, exhibiting a prevalence rate of approximately 4% across various tumor types, as illustrated in Figure 3A and corroborated by multiple studies [52,53,54,55,56,57]. Notably, the incidence of *MET* amplification surges to nearly 20% in kidney papillary cell carcinomas (KIRP), a data point presented in Figure 3B. Moreover, *MET* amplification has been shown to confer resistance to therapies targeting the epidermal growth factor receptor (EGFR) in malignancies such as NSCLC and colorectal cancer, underlining its role in both cell survival and acquired resistance to EGFR-targeting therapies [56,58,59].

While interpreting these findings, it is crucial to place MET amplification in the broader context of common chromosomal aberrations in cancer, such as cellular aneuploidy. Specifically, the trisomy of chromosome 7 is often observed across multiple cancer types and serves as a pan-cancer genetic marker [60,61,62]. This trisomy could confound the assessment of *MET* amplification because both are related, but have distinct genomic alterations affecting cellular phenotype and treatment response. Unlike chromosome 7 trisomy, which is not a primary cancer driver, *MET* amplification acts as a driver and represents a true biological selection [3]. In vitro and preclinical studies suggest that a ‘threshold’ of five copies of the *MET* gene drives addiction, thus justifying targeted therapies [63]. Although no clinical consensus exists for such a cut-off, it is critical for effective patient stratification in MET-targeted therapies. Fluorescence in situ hybridization (FISH) techniques can distinguish between chromosome 7 polysomy and true *MET* amplification. In the case of polysomy, the *MET*-to-centromere of the chromosome 7 ratio (*MET*/*CEN7*) remains constant, whereas it increases for biologically selected true *MET* amplification, identifying a patient subgroup that could benefit from targeted therapies. Current advances in next-generation sequencing (NGS) techniques can also offer invaluable information for better patient stratification. Indeed, recent studies have demonstrated the efficiency of MET inhibition when patients were classified using either the *MET*/*CEN7* ratio or NGS-based detection of the *MET* copy number, as shown in Table 1 and supported by studies [64,65].

## 5. *Exon14* ‘Skipping’, the Predominant *MET* Alteration

It was thus proposed that other mutations might drive ligand-independent activation of MET. Surprisingly, for an RTK (e.g., *EGFR*, fibroblast growth factor receptor (*FGFR*), for review, see [105]), very few patients exhibited *MET* mutations in the kinase domain or regulatory regions (Figure 3C and [17,106]). Splice site mutations spanning the *Exon14* were by far the most common *MET* mutations. These mutations (complex or simple) are a consequence of the loss of acceptor or donor sites, resulting in *Exon14* ‘skipping’. Indeed, the latter was described in a significant number of patients: 13% in pulmonary sarcomatoid carcinoma, 6% in adenosquamous carcinoma, 3% in lung adenocarcinomas, 2% in lung squamous cell carcinomas, 0.4% in gliomas, and 0.4% in cancers of unknown primary origin (CUP) [16,107]. *MET Exon14* encodes for the juxtamembrane domain. Because of the regulatory role of the JM described above, it was thought that its loss would lead to increased receptors, and the subsequent ligand-independent uncontrolled activation of MET, thus driving invasive growth. Accordingly, the re-insertion of *Exon14* into the oncogenic gene fusion (*TPR*)–*MET*, which consists of the *MET* sequence downstream from the juxtamembrane domain fused to the dimerization motif of *TPR*, resulted in decreased oncogenic potential [108]. Therefore, targeted therapies against MET constituted appealing strategies for cancer patients carrying *MET Exon14* deletion (*MET∆14*) [21,23,72,79,84,109,110,111,112,113,114,115]. However, only half of the patients harboring MET∆14 benefited from MET-targeted therapies, suggesting that the critical aspects of MET∆14 remain to be elucidated [116,117]. Recently, two independent studies demonstrated that the deletion of *Exon14* does not result in constitutive activation of the kinase. MET∆14 activity requires SF and drives a robust and selective AKT activation, rendering cancer cells more prone to survival and migration [17,118]. Therefore, it is proposed that cancer cells expressing MET∆14 choose the astute strategy to ‘fly’ the local hostile micro milieu to form distal metastases instead of ‘fighting’ to proliferate locally. The absence of SF in the tumor microenvironment or PI3K/AKT axis mutations may explain the insensitivity to targeted therapies. Fittingly, PI3K/AKT activating mutations co-occur with *MET Exon14* ‘skipping’ in 14% of cancer patients [119,120]. Based on these results, a better stratification of patients might lead to a better response to MET-targeted therapies.

## 6. Point Mutations within the *MET* Coding Sequence

As shown in Figure 3C, mutations affecting the catalytic site or regulatory sites of *MET* are sporadic but do exist. The first activating mutations of *MET* were identified in hereditary papillary renal carcinoma (HPRC), and the authors suggested that the mutations affecting the kinase domain of MET (M1149T, V1206L, V1238I, D1246N, and Y1248C) were causal in HPRC [121]. Similar *MET* mutations (D1246H, Y1228C, and M1268T) spanning the critical Y1234 and Y1235 were described in the sporadic renal carcinoma [122]. Moreover, cytogenetic analyses showed non-random chromosome 7 trisomy, which affected the mutated *MET* allele [123]. All these mutations have the common feature of inducing the constitutive activation of the kinase [122,124,125], leading to oncogene ‘addiction’ [3]. Experiments in transgenic murine demonstrated the oncogenic potential of these activating mutants.

Interestingly, tumors formed in mice were not restricted to the kidney; animals developed lymphomas, carcinomas, or aggressive mammary tumors [126,127]. Importantly, these independent studies demonstrate that activating mutations affecting the MET catalytic site drive tumorigenesis in multiple tissues. Researchers attempted to identify activating mutations in other human cancers in the following years. Somatic or germline mutations were described in hepatocellular carcinoma, head and neck cancers, oropharynx squamous cell cancer, gastric cancer, cancers of unknown primary origin (CUP), and colorectal cancer [128,129,130,131,132,133,134]. 

A growing number of point mutations were described in the SEMA domain (both in *α* and *β* chains, Figure 3C) responsible for the ligand binding [135]. Six non-small cell lung cancer patients out of 127 harbored mutations (L229F, N375S, E168D, N375S, S323G, N375S) within the *Exon2* encoding for the SEMA domain [136]. SEMA domain mutations are not restricted to lung cancer. In an independent study, *MET* mutations were detected in 9% of advanced breast cancer (8/88 patients). Six of eight *MET* mutations affected the SEMA domain (N375S in five patients, M362T in one patient, [137]). Despite some evidence showing that SEMA domain mutations are oncogenic [133,138], our knowledge of the biological significance of *MET* mutations affecting the SEMA domain remains poor. They likely affect the ligand-binding domain’s structure, promoting a constitutively active or ligand-hypersensitive kinase. Accordingly, in CUP—owning the unique ability of self-renewal in the absence of any exogenous growth factor [139]—*MET* mutations were clustered within the SEMA domain; in a total of 23 CUP patients, five out of seven MET alterations were localized in the SEMA domain (H150Y, Q142X, C385Y, and two patients with E168D [133]). 

Point mutations, although rare, should not be overlooked. With recent advancements in genomic screening technologies, we might find a growing number of these mutations. By combining in silico, in vitro, and in vivo findings, we aim to have a sharper picture of *MET* alterations in cancer and their biological significance, opening new avenues for targeted therapies.

For RTKs, it is generally accepted that gene amplification leads to a higher number of receptors at the cell surface, priming kinase activation in the presence of small amounts of ligands. MET challenges this concept; an overexpression of human *MET* in mouse liver induces hepatocellular carcinomas [140]. Since human MET cannot interact with murine SF [141], these results demonstrate the ligand-independent activation of the receptor when overexpressed. 

## 7. Fusion Partners of *MET* Drive Oncogene ‘Addiction’

For decades, the only known *MET* gene rearrangement in human tumors has been *TPR*-*MET*, mostly occurring in gastric cancers [142]. Recently, the thorough analyses of the vast TCGA tumor collection uncovered new hybrid proteins [143]: the MET intracellular domain fused at the N-terminus with several partners, some of them encompass the dimerization ‘coiled-coil’ (CC) motif (i.e., C8orf34, BAIAP2L1, TFG, and KIF5B). Consequently, the chimeric MET dimerizes in a ligand-independent fashion, driving constitutive kinase activity and tumorigenesis. Although occurring at low frequencies, these fusions have been found in lung adenocarcinomas, hepatocellular carcinomas, papillary renal carcinomas, and thyroid carcinomas; thus, they cannot be ignored [143].

Another recurrent gene rearrangement involves *MET* and the *PTPRZ1* gene, encoding a tyrosine phosphatase [144]. *PTPRZ1–MET* fusions include almost the entire MET sequence fused at its 5′ end with a variable number of *exons* of the *PTPRZ1* gene [145]. *PTPRZ1*-*MET* fusions have been found in brain tumors, such as low-grade gliomas, secondary glioblastomas arising in adults from the progression of lower-grade gliomas, and pediatric glioblastomas at a remarkably high frequency (~10%, [145]). Notably, the chromosomal rearrangement between *PTPRZ1* and *MET* leads to fusion protein overexpression and enhanced kinase activation [146]. The mechanism explaining enhanced MET activity in tumors expressing the fusion protein remains to be determined; the highly active *PTPRZ1* promoter fused to the *MET* gene [145] and the coiled-coil domain of *PTPRZ1* fused to *MET* [147] are two mutually non-exclusive hypotheses.

Experiments show that MET fusion proteins respond to anti-MET monotherapy: *PTPRZ1-MET* in a pediatric glioma [145] and *KIF5B-MET* in lung cancers [148]. *MET* gene fusions also happen in melanomas, where six different N-terminal partners fused in-frame with the intracellular MET domain have been described [23]. 

**Figure 4:**
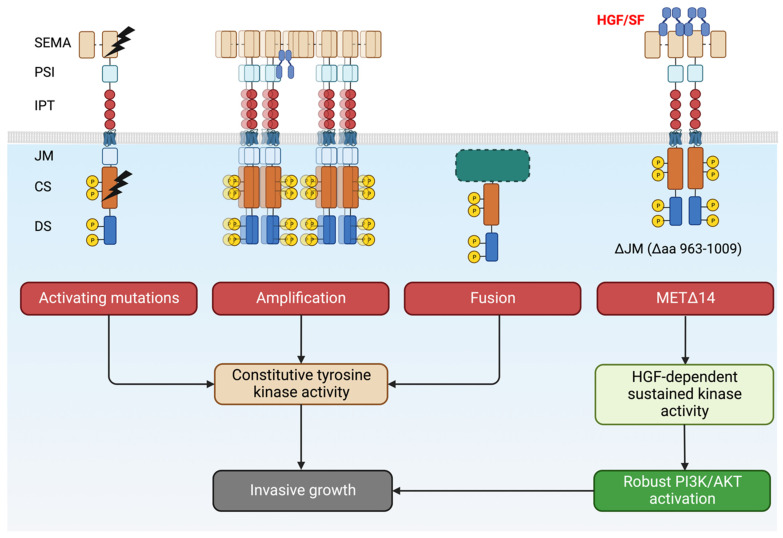
Overview of *MET* alterations in cancer that drives invasive growth. Tumors become ‘addicted’ to MET and become a candidate for targeted therapies. Created with BioRender.com (accessed on 5 August 2023).

Different *MET* genetic alterations can induce either ligand-independent (or hypersensitive) or SF-dependent (MET∆14) activation of the kinase (Figure 4). They have a common denominator for driving invasive growth. Tumor cells thus become addicted to MET and become vulnerable to targeted therapies. 

## 8. Genetic Alterations of *MET*: The Peak of the Iceberg?

*MET*-amplified tumors represent 4%, where MET overexpression is observed in more than 50% of cancers (Figure 3D). The discrepancy is too large and deserves the attention of the scientific community. It should be noted that a large dataset often restricts their analyses to protein-coding regions, overlooking important regulatory regions crucial for gene expression. The inducible nature of the *MET* promoter was previously described and its importance in tumor biology was established [149,150,151]. Thus, alterations affecting the *MET* promoter should not be disregarded: re-analyses of TCGA dataset showed a remarkable decrease in promoter methylation in cancer patients, a synonym for transcription activation and *MET* overexpression (Figure 3E). This observation suggests that central regulatory mechanisms remain to be elucidated. Gene expression is not only regulated at the transcription level. Post-transcription control mechanisms affecting the translation efficiency and the messenger’s stability are hubs for deregulations observed in cancer. Indeed, we observed a small but significant fraction of patients harboring mutations within untranslated regions of the mRNA (Figure 3F). However, despite their biological significance being unknown, their exploration might reveal an unsuspected ‘dark energy’ for tumor cells. Additionally, we have recently revealed *MET* translational regulation by the PI3K/AKT/mTOR axis and its relevance in therapy resistance. This aspect is detailed below.

## 9. MET-Targeted Therapies

Several MET-targeting agents have been developed in line with the statements above (summarized in Table 1). As with other targeted therapies, several questions must be addressed before trying to quench MET signaling and tumor growth in patients. We can encapsulate these matters in three main topics: (1) how to quench MET signaling in patients; (2) who should benefit from MET-targeting agents; and (3) how to face the inevitable problem in the targeted therapy: drug resistance. 

## 10. Different MET-Blocking Agents: Advantages and Pitfalls

**Figure 5:**
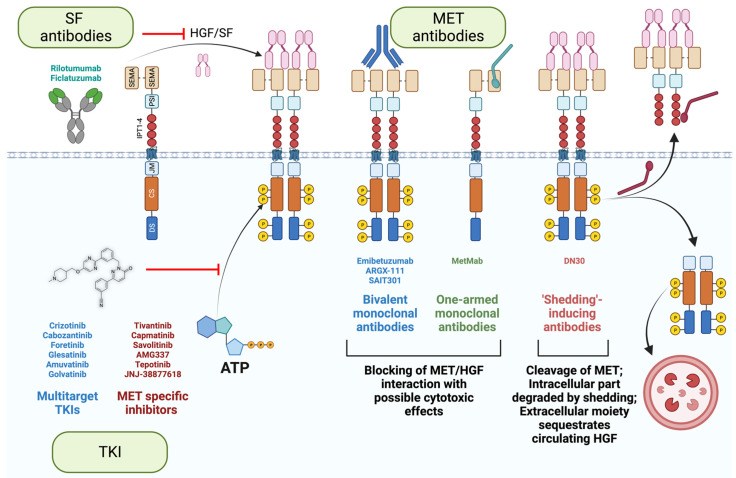
Targeting MET/SF axis in cancer. Tyrosine kinase inhibitors (TKI) act as ATP mimetics to impede receptor phosphorylation. MET/SF interaction using can be prevented using either SF antibodies or MET antibodies. Some MET antibodies can induce receptor degradation in CBL-independent manner (SAIT301) or by shedding (DN30). Additionally, in the case of DN30, the extracellular part of the trimmed receptor has the potential to sequestrate circulating SF. Created with BioRender.com (accessed on 5 August 2023).

Three main strategies were employed to extinguish the MET signaling (schematized in Figure 5). Firstly, small kinase inhibitors and monoclonal antibodies targeting SF or its receptor [3,12,152]. Small kinase inhibitors are chemical compounds that pass through the plasma membrane and interact with the receptor kinase domain. They can target a large panel of receptors (multitarget tyrosine kinase inhibitors), specifically MET. The latter has the advantage of reducing off-target effects. On the other hand, because of the crosstalk of receptors [44,153,154,155], targeting a broader number of RTKs might induce a better clinical outcome. Tyrosine kinase inhibitors act as ATP mimetics, hampering receptor phosphorylation and subsequent kinase activity [156]. However, as discussed in the first chapter, MET phosphorylation is indispensable for its degradation [36,37]. In the chronic treatment setting, small molecules can thus potentially increase the number of receptors at the cell surface, suffocating the treatment efficacy. Additionally, an acquired, or existing mutation in the ATP binding pocket can engender resistance, as previously observed for small molecule inhibitors of EGFR, KIT, and BCR-ABL [157,158,159,160]. 

Secondly, an alternative approach is to target the extracellular moiety of MET using antibodies. They are more specific by nature than chemical inhibitors. Importantly, antibodies are insensitive to multidrug resistance, a feature of aggressive cancer cells that augments the drug efflux, reduces its influx, or increases the drug catabolism [56,161,162,163,164]. Furthermore, antibodies recognize MET, even if the intracellular part is mutated in cancer cells (METΔ14 or activating mutations in the catalytic site). However, antibodies can induce receptor dimerization and activation. Different strategies have been employed to circumvent this issue. One-armed monoclonal antibodies (MetMab, also known as Onartuzumab, [90]) and antibodies inducing CBL-independent degradation of MET (SAIT301, [97,98]) have been developed and tested in clinics (Table 1). 

Lastly, another innovative strategy is taking advantage of the shedding of the plasma membrane to maintain homeostasis [165]; DN30 antibodies interact with the IPT domain of MET with subnanomolar affinities and induce shedding through the cleavage of the extracellular moiety of MET [166,167,168]. The proteasome subsequently degrades the intracellular moiety [169,170]. Additionally, since the extracellular part of MET is trimmed, it can potentially sequestrate the circulating SF, further hampering the SF/MET axis. Furthermore, based on the straightforward elimination of MET from the cell surface, independently of receptor activation (phosphorylated vs. unphosphorylated state), DN30 has a substantial advantage over other MET antibodies, as it is effective in the full spectrum of MET activation mechanisms, whether SF-dependent or independent (that is, induced by mutations, gene fusions, or amplification). Multiple studies demonstrated its remarkable potential; DN30 hampers cell growth and induces apoptosis in multiple MET-addicted cell lines in vitro, and induces an impressive reduction of tumor mass in vivo [168,171]. Importantly, DN30 displayed a favorable pharmacokinetics and safety profile in non-human primates [171], encouraging the design of a clinical trial in MET-addicted cancer patients.

Targeting the ligand is an alternative option to impede MET signaling. The benefit of the SF antibodies AMG 102 (Rilotumumab, [101]) and AV-299 (Ficlatuzumab) was assessed in many clinical trials with disappointing results (Table 1). In one clinical trial, SF antibodies significantly increased mortality, causing the premature ending of the study [99]. As discussed previously, *MET* alterations in tumors typically promote ligand-independent kinase activity (gene amplification, activating mutations, fusion proteins), partially explaining these frustrating results. Nevertheless, targeting SF should not be dismissed; their ability to sequestrate the ligand could be exploited to inhibit SF-dependent invasion and survival in tumors without *MET* amplification (e.g., METΔ14). Furthermore, it should be reminded that SF acts on the tumor micro milieu on cancer-associated fibroblasts and macrophages to foster angiogenesis [172,173,174,175]; therefore, a strategy is valid only if employed toward the right target.

## 11. Patient Stratification: A Key for Success in Targeted Therapies

Identifying the target population for treatment might look self-evident, but it is laborious and critical for setting the scene for success in a clinical trial. More than three decades of basic research guided drawing an overall picture of patients that can benefit from MET inhibition. Studies using cell lines or patient-derived xenografts have shown that only tumors harboring *MET* alterations (mostly amplification) respond to the MET blockade. The cell cycle is arrested, and/or apoptosis is induced in vitro [176], and complete inhibition of tumor growth (and even tumor shrinkage) is observed in vivo [59]. *MET* alterations must be assessed in patients before assigning them to the group receiving targeted therapies. This is indeed a golden rule to follow, as targeted therapies can only benefit patients presenting *MET* alterations. Nevertheless, their unambiguous identification is challenging. In many clinical trials, MET levels were assessed by immunohistochemistry [83,92], a strategy being far from objective and hardly reproducible. Moreover, the increased protein intensity is not necessarily a synonym for *MET* amplification and ‘addiction’. Because of the inducible nature of the *MET* promoter [149,150,151], high-MET-protein levels may be transient due to changes in the tumor microenvironment (e.g., hypoxia, ionizing radiation, cytotoxic reagents, described as ‘expedience’ in [3]). Indeed, post hoc analyses of some clinical trials illustrate this issue; patients with a gene copy number gain of MET > 4 exhibited the uppermost progression-free survival [92]. Another study testing the efficiency of MetMab failed to demonstrate any benefit of the treatment, although 88% of patients were MET-negative [87], demonstrating abruptly the validity of the golden rule stated above. 

For tumors expressing wt *MET* (the vast majority of patients), a priori ineligible for MET-targeted therapies, it should be noted that hampering the MET signaling reduces migration and metastatic dissemination drastically without affecting the growth of cancer cells [63,177,178]. Using MET-targeted therapy might constitute a promising adjuvant therapy after tumor resection with curative intent, an ideal setting to eradicate the persistence and dissemination of subclinical tumor foci.

With the progress in next-generation sequencing technologies (lesser quantity of material required for acceptable coverage), genomic interrogations of liquid biopsies (circulating tumor DNA and circulating tumor cells) are largely feasible [179,180] to adequately and objectively stratify patients. Ideally, *MET* alterations must be assessed before the initiation of the clinical trial. Liquid biopsies also enable longitudinal evaluation of tumor evolution in a non-invasive manner, crucial for understanding mechanisms hidebound to drug resistance, the major challenge in targeted therapy.

## 12. Understanding and Overcoming Drug Resistance

Targeted therapies were a breakthrough in cancer research, emphasizing our cutting-edge understanding of cancer cells’ vulnerabilities. The design of appropriate treatment strategies was thus achievable. However, they suffer a significant limitation: resistance frequently occurs [181,182,183] after an initial response. Understanding how cancer cells evade targeted therapies constitutes a substantial challenge in the clinic.

Tumors can be defined as pseudo-organs constituted of heterogeneous clones presenting highly diverse genotypes and phenotypes. Some might prosper while others regress depending on their ability to face microenvironmental selection pressures [183]. *MET* genetic alterations might dominate the majority of tumor cells and dictate drug sensitivity; however, minor subclones harboring other mutations that confer resistance to *MET* blockade may coexist and be positively selected under drug pressure. Resistant subclones must be promptly detected (e.g., liquid biopsies at regular intervals). In line with this idea, many efforts have been made to characterize the molecular profile of emerging resistant clones [184,185,186]. These forerunner studies have undeniable value for discovering new targetable biomarkers in persistent clones that limit their propagation with proper and timely therapeutic interventions. 

## 13. The Flare Effect: mTOR Pathway Comes to Scene

In current clinical practice, the line of treatment is dismissed when resistance arises. In many patients, discontinuation of kinase inhibitors results in rapid tumor regrowth: this phenomenon is known as disease ‘flare’ or tumor ‘rebound’ [187,188,189,190], characterized by an unknown incidence and a cumbersome prognosis [191]. The occurrence of tumor ‘rebound’ complicates disease management and has led to the idea of continuing the therapy beyond the progression [191]. Notably, treatment with a MET therapeutic antibody that induces ‘shedding’ (proteolytic cleavage of the receptor at the cell surface) substantially prevents this effect, providing a rationale to combine, or alternate, MET-targeted drugs with different mechanisms of action [192]. 

We recently presented a mechanistic elucidation of the ‘flare’ effect [193]. Within MET-amplified cells, halting the administration of the small molecule JNJ-38877618 (MET inhibitor) triggered activation within the protein kinase B/mechanistic target of the rapamycin (AKT/mTOR) pathway. Notably, mTOR orchestrates cell growth by governing mRNA translation, ribosome biogenesis, and metabolic processes [194,195,196,197]. Noteworthy transformations have been observed due to the overexpression of mTOR’s target, eIF4E, in rodent fibroblasts [198], consequently prompting extensive investigations into mTOR’s role in cancer [194]. Efforts towards inhibiting mTOR with rapalogs (including rapamycin and analogs) have been pursued in cancer therapeutics; however, the exclusive utilization of rapalogs displayed suboptimal efficacy [199].

A captivating observation emerged as mTOR swiftly augmented *MET* translation upon MET inhibitor withdrawal [193], uncovering a novel aspect of the AKT/mTOR axis in conferring resistance against therapies. Beyond de novo protein synthesis, the influence of the AKT/mTOR pathway on MET extends further. This pathway’s Ser/Thr kinase activity phosphorylates and incapacitates the protein phosphatase PTP1B, resulting in elevated phosphorylated (active) MET at the cell surface [193]. Consequently, the AKT/mTOR pathway emerges as a promising candidate for targeted interventions due to its paramount role in driving tumor rebound. These mechanistic insights hold the potential to guide the formulation of metronomic treatment strategies, incorporating alternating MET inhibitors and mTOR inhibitors with minimal washout periods.

In a broader context, the intricate interplay between the mTOR axis and MET biology underscores the pivotal role of translational regulation in governing MET expression within the realm of cancer. Translational control, distinct from the transcriptional regulations, operates on pre-existing mRNA, facilitating swift adaptability to the dynamic shifts within the tumor microenvironment. While a comprehensive evaluation of the relevance of *MET*’s translational control in the context of cancer demands further exploration, it might provide insight into the apparent paradox between sporadic MET amplifications (accounting for 3–5% of cases) and the nearly ubiquitous MET overexpression observed in cancer [200,201,202,203].

## 14. Conclusions

MET is a potent oncogene driving invasive growth [3,13,14,15,16,17]. Decades of fundamental research led to understanding MET’s different roles in human tumors. New studies are ongoing to fine-tune our understanding of the role of varying *MET* mutations in cancer [17,118,138]. The biological knowledge of MET needs to be translated into clinical applications. Recent failures of clinical trials can be primarily explained by the lack of consideration of *MET* genetic alterations before patient selection. On the contrary, when patient stratification was appropriately performed a priori or during post hoc analyses, a general picture of the target patients’ genomic profile can be pictured: *MET* alterations are an absolute prerequisite for the success of targeted therapies. 

These lessons bear witness to crucial importance of the functional preclinical insights in guiding clinical practice. Indeed, it was already clear from studies in cell lines and animal models that substantial reduction of cancer cell viability in vitro and tumor shrinkage in vivo occur only in settings where stable and heritable genetic alterations of *MET* sustain oncogene ‘addiction’. For the large majority of cancer patients, where *MET* is not mutated, preclinical comprehension of the role of MET can be directly translated to the clinic: MET has anti-apoptotic and pro-invasive functions [177,178]. Adjuvant treatment with MET inhibitors after tumor resection with curative intent could be a viable strategy.

Another unexplained phenomenon is the relatively poor frequency of MET-amplified tumors [16,52,53,54,55,56,57,107], compared to many MET-overexpressing tumors [200,201,202,203]. This apparent paradox can be partially explained by the inducible nature of the *MET* promoter: ionizing radiations, hypoxia, and cytotoxic reagents enhance MET expression [149,150,151], previously described as oncogene-mediated ‘expedience’ [3]. Translational control mechanisms (e.g., mTOR) might add another layer of regulation of the receptor expression [193]. Another possibility is the existence of other mutations, either in the coding sequence or regulatory regions of the *MET* gene. The number of previously unidentified *MET* mutations is constantly increasing thanks to the advances in next-generation sequencing techniques and comprehensive publicly available databases (TCGA, cBioPortal, ClinVar). Thus, efforts aiming at the identification of new variants in cancer must be encouraged. The primary task should be to functionally characterize these mutations to broaden the panel of MET ‘addicted’ tumors and increase the number of patients eligible for next-generation precision medicine. 

## Figures and Tables

**Table 1:** * As described in August 2022 in clinicaltrials.gov. Early phase I: exploratory trials before phase I. NA: trials without FDA-defined phases. ** Advanced cancers of various origins; ADCC: antibody-dependent cell-mediated cytotoxicity; AML: acute myeloid lymphoma; HCC: hepatocellular carcinoma; NSCLC: non-small cell lung cancer; PRCC: papillary renal cell carcinoma; SCLC: small cell lung cancer.

Drug	Number of Trials (Phase) *	Cancer Types	Principal Outcome	Notes
Multitarget tyrosine kinase inhibitors				
PF02341066 (Crizotinib)	51 (early I/I); 62 (II); 18 (III); 5 (IV); 7 (NA)	Breast cancer, renal clear cell cancer, glioblastoma, inflammatory myofibroblastic tumors, lymphoma, papillary renal cancers, MET+ gastric adenocarcinoma, MET+ or RON+ metastatic urothelial cancer and NSCLC	Substantial antitumor activity in patients with *MET* amplification and/or METΔ14 [66,67,68]. Crizotinib overcomes resistance to selpercatinib in *RET*-fusion positive NSCLC patients [69].	Targets: MET, ROS1, and ALK Approved for treating NSCLC with EML4–ALK in 2011 and NSCLC with CD74–ROS1 in 2016 [67].
XL184 (Cabozantinib)	54 (early I/I); 157 (II); 19 (III); 2 (IV); 7 (NA)	Breast cancer, glioblastoma, HCC, kidney cancer, medullary thyroid cancer, melanoma, NSCLC, ovarian cancer, and prostate cancer	Cabozantinib significantly improved progression-free survival in patients with metastatic PRCC and melanoma [70,71]. Complete response was reported in one patient with METΔ14 [72].	Targets: MET, RET, and others. Approved for the treatment of medullary thyroid cancer [73].
GSK1363089 (Foretinib)	6 (early I/I); 7 (II)	Mixed cancer, breast cancer, gastric cancer, head and neck cancer, liver cancer, NSCLC, and PRCC	No activity in unselected patients [74].	Targets: MET, RET, and others.
MGCD265 (Glesatinib)	4 (early I/I); 2 (II)	Mixed cancer and NSCLC	Results are pending. The safety profile is acceptable in non-genetically selected patients with advanced solid tumors [75].	Targets: MET and AXL
MP470 (Amuvatinib)	2 (early I/I); 1 (II); 1 (NA)	Mixed cancer, gastric cancer, glioblastoma, pancreatic cancer, and SCLC	Results are pending.	Targets: MET, RET, FLT3, and PDGFRA
E7050 (Golvatinib)	8 (early I/I)	Mixed cancer, gastric cancer, head and neck cancer, and HCC	Results are pending.	Targets: MET and VEGFR2
Specific MET inhibitors (small molecules)				
ARQ197 (Tivantinib)	25 (early I/I); 21 (II); 4 (III)	Mixed cancer **, colorectal cancer, HCC, liver cancer, mesothelioma, NSCLC, SCLC, and stomach cancer	Tivantinib treatment did not demonstrate efficacy in a Phase III trial for HCC patients with high MET levels (based on staining intensity) [76].	Specificity to MET is controversial [77,78].
INCB28060 (Capmatinib)	18 (early I/I); 27 (II); 3 (III); 1 (IV)	Mixed cancer, colorectal cancer, glioblastoma, head and neck cancer, HCC, NSCLC, and PRCC	Capmatinib showed substantial antitumor activity in patients with MET amplification or METΔ14 [79,80,81]. Capmatinib induces potentially similar resistance mechanisms as Crizotinib [82] but is a promising option in MET-amplified, EGFR-inhibitor-resistant tumors [80].	Approved to treat adult patients with metastatic NSCLC with METΔ14 [83].
AZD6094 (Savolitinib or Volitinib)	7 (early I/I); 8 (II); 3 (III)	Mixed cancer, colorectal cancer, gastric cancer, NSCLC, kidney cancer, and PRCC	Encouraging results in EGFR-mutated, MET-amplified tumors [64,65].	NA
AMG337	3 (early I/I); 5 (II)	Mixed cancer, renal clear cell cancer, esophageal cancer, and stomach cancer	Results are pending.	NA
MSC2156119J (Tepotinib)	5 (early I/I); 5 (II)	Mixed cancer, lung cancer, and NSCLC	Partial response in NSCLC patients with METΔ14 [84]. Promising results in patients with *MET* amplification [85,86].	NA
OMO-1 (JNJ-38877618)	1 (early I/I)	Mixed cancer, lung cancer, and NSCLC	Results are pending.	NA
MET antibodies				
MetMab (Onartuzumab)	6 (early I/I); 8 (II); 5 (III)	Mixed cancer, breast cancer, colorectal cancer, glioblastoma, HER2- and MET+ gastric cancer, HCC, and MET+ NSCLC	Some clinical trials were inconclusive due to poor patient selection or the premature termination of the study [87,88,89]. Other results are pending.	One-armed monoclonal antibody [90].
LY2875358 (Emibetuzumab)	1 (early I/I); 2 (II)	Mixed cancer, gastric cancer, and NSCLC	Cytostatic antitumor activity [91]. Significant increase in median progression-free survival for patients with the highest MET expression [92]. It cannot reverse acquired resistance to Erlotinib, an EGFR inhibitor [93].	Humanized IgG4 bivalent monoclonal antibody [94].
ARGX-111	1 (early I/I)	Mixed cancer, gastric cancer, glioblastoma, liver cancer, and renal cancer	Results are pending.	Bivalent monoclonal antibody with the property to activate ADCC [95].
SAIT301	1 (early I/I)	Mixed cancer	Phase I completed, and the recommended dose for phase II was established [96].	Bivalent monoclonal antibodies targeted against the MET α chain, inducing CBL-independent degradation of MET to circumvent the detrimental agonist effect of other bivalent antibodies [97,98].
SF antibodies				
AMG 102 (Rilotumumab)	6 (early I/I); 13 (II); 3 (III)	Mixed cancer, gastric cancer, glioblastoma, lung cancer, mesothelioma, and prostate cancer	No improvement in the clinical outcome in patients with MET+ gastric cancer [99]. Toxicity issue [100].	Humanized IgG2 monoclonal antibody [101].
AV-299 (Ficlatuzumab)	6 (early I/I); 4 (II); 3 (III)	AML, head and neck cancer, liver cancer, and NSCLC	Low benefit compared to other drugs [102,103].	Humanized IgG1 monoclonal antibody [104].

## Data Availability

Not applicable.
